# Prevalence of endocrine and genetic abnormalities in boys evaluated systematically for a disorder of sex development

**DOI:** 10.1093/humrep/dex280

**Published:** 2017-08-30

**Authors:** R. Nixon, V. Cerqueira, A. Kyriakou, A. Lucas-Herald, J. McNeilly, M. McMillan, A.I. Purvis, E.S. Tobias, R. McGowan, S.F. Ahmed

**Affiliations:** 1 Developmental Endocrinology Research Group, University of Glasgow, Royal Hospital for Children, Office Block, 1345 Govan Road, Glasgow G51 4TF, UK; 2 West of Scotland Clinical Genetics Service, Queen Elizabeth University Hospital, Glasgow G51 4TF, UK; 3 Biochemistry Department, Queen Elizabeth University Hospital, Glasgow G51 4TF, UK; 4 Academic Medical Genetics and Pathology, University of Glasgow, Queen Elizabeth University Hospital, Glasgow G51 4TF, UK

**Keywords:** copy number variant, aetiology, disorder of sex development, XY DSD, genitalia, endocrine abnormaility, genetic abnormality

## Abstract

**STUDY QUESTION:**

What is the likelihood of identifying genetic or endocrine abnormalities in a group of boys with 46, XY who present to a specialist clinic with a suspected disorder of sex development (DSD)?

**SUMMARY ANSWER:**

An endocrine abnormality of the gonadal axis may be present in a quarter of cases and copy number variants (CNVs) or single gene variants may be present in about half of the cases.

**WHAT IS KNOWN ALREADY:**

Evaluation of 46, XY DSD requires a combination of endocrine and genetic tests but the prevalence of these abnormalities in a sufficiently large group of boys presenting to one specialist multidisciplinary service is unclear.

**STUDY, DESIGN, SIZE, DURATION:**

This study was a retrospective review of investigations performed on 122 boys.

**PARTICIPANTS/MATERIALS, SETTING, METHODS:**

All boys who attended the Glasgow DSD clinic, between 2010 and 2015 were included in the study. The median external masculinization score (EMS) of this group was 9 (range 1–11). Details of phenotype, endocrine and genetic investigations were obtained from case records.

**MAIN RESULTS AND THE ROLE OF CHANCE:**

An endocrine abnormality of gonadal function was present in 28 (23%) with a median EMS of 8.3 (1–10.5) whilst the median EMS of boys with normal endocrine investigations was 9 (1.5–11) (*P* = 0.03). Endocrine abnormalities included a disorder of gonadal development in 19 (16%), LH deficiency in 5 (4%) and a disorder of androgen synthesis in 4 (3%) boys. Of 43 cases who had array-comparative genomic hybridization (array-CGH), CNVs were reported in 13 (30%) with a median EMS of 8.5 (1.5–11). Candidate gene analysis using a limited seven-gene panel in 64 boys identified variants in 9 (14%) with a median EMS of 8 (1–9). Of the 21 boys with a genetic abnormality, 11 (52%) had normal endocrine investigations.

**LIMITATIONS, REASONS FOR CAUTION:**

A selection bias for performing array-CGH in cases with multiple congenital malformations may have led to a high yield of CNVs. It is also possible that the yield of single gene variants may have been higher than reported if the investigators had used a more extended gene panel.

**WIDER IMPLICATIONS OF THE FINDINGS:**

The lack of a clear association between the extent of under-masculinization and presence of endocrine and genetic abnormalities suggests a role for parallel endocrine and genetic investigations in cases of suspected XY DSD.

**STUDY FUNDING/COMPETING INTEREST(S):**

RN was supported by the James Paterson Bursary and the Glasgow Children's Hospital Charity Summer Scholarship. SFA, RM and EST are supported by a Scottish Executive Health Department grant 74250/1 for the Scottish Genomes Partnership. EST is also supported by MRC/EPSRC Molecular Pathology Node and Wellcome Trust ISSF funding. There are no conflicts of interest.

**TRIAL REGISTRATION NUMBER:**

None.

## Introduction

A disorder of sex development (DSD) can present as a range of phenotypes, from slightly atypical genitalia to complete sex reversal and despite advances in our understanding of the processes of sex determination and differentiation, the aetiology of 46, XY DSD remains elusive in several cases (Ahmed & Rodie, 2010). Reaching a definitive diagnosis is important in cases of DSD as it can enable genetic counselling to address issues such as aetiology andrecurrence risk for individuals and their families and it may also clarify management relating to adrenal health, gonadal tumour risk and long-term fertility (Kyriakou *et al.*, 2015). An aetiological diagnosis was reported in 31% of cases of severe hypospadias in 2001 (Boehmer *et al.*, 2001) and, when using the DSD classification that was recommended a decade ago (Hughes *et al.*, 2006), a genetic diagnosis has been reported to be clear in over 80% of boys with a disorder of androgen synthesis (DAS; Morel *et al.*, 2011), about 40% of boys with a disorder of gonadal development (DGD; Laino *et al.*, 2014) and probably about 20% of boys with a suspected disorder of androgen action (Ahmed *et al.*, 2000a). In addition, 30% of DSD cases may have copy number variants (CNVs) on array-comparative genomic hybridization (array-CGH; Baetens *et al.*, 2014). However, the extent of investigations performed and the selection of cases that undergo investigations may vary extensively from one centre to another (Rodie *et al.*, 2011) and the yield of abnormalities may depend on the approach adopted by the centre. The aim of the current study was to gain an insight into the prevalence of genetic and endocrine abnormalities in a contemporary cohort of 46, XY DSD boys in the UK who were investigated systematically according to recommendations of a recent expert group (Ahmed *et al.*, 2016) at one specialist centre. In addition, information was collected to explore any association between the clinical features of these boys and the results from endocrine and genetic investigations.

## Methods

### Patients

All boys with a confirmed or presumed 46, XY karyotype who were reviewed for atypical genitalia by the Glasgow DSD clinic between January 2010 and December 2015 were included in the analysis. Boys with isolated unilateral undescended testis on clinical examination were excluded. Detailed phenotypic information was recorded to calculate an external masculinization score (EMS) (Ahmed *et al.*, 2000b) to objectively document the degree of masculinization of the infant's genitalia.

### Endocrine analysis

The following hormone levels were measured at baseline or following hCG stimulation test: anti-Müllerian Hormone (AMH) using Beckman Gen 11 ELISA, Androstenedione (A) and Testosterone (T) by LC MS/MS using Water Xevo TMS and Dihydrotestosterone (DHT) by LC/MS/MS using a Waters mass spectrometer. FSH and LH were measured on the Abbott Architect ci1600 using chemiluminescent microparticle immunoassays (Abbott Laboratories Diagnostics, Santa Clara, CA, USA). The hCG stimulation test was performed as described previously (Dixon *et al.*, 2007) where 1500 units of intramuscular hCG was administered on three consecutive days followed by testosterone, dihydrotestosterone and androstenedione levels being recorded on Day 4. For some children this was followed by hCG stimulation using 1500 units on 2 days of the week over a 2-week period with testosterone levels being measured on Day 22. Testosterone response was reported as normal if the absolute testosterone concentration level increased by two or more times the baseline, or was above the upper limit of prepubertal range (Ahmed *et al.*, 2016).

### Genetic analysis

DNA was extracted from peripheral blood samples using standard methods for patients undergoing routine clinical genetic testing. Chromosomal abnormality was identified by karyotypes or array-CGH. Analysis of seven DSD associated genes including *SRY, AR, NR5A1, HSD17B3, MAMLD1, NR0B1* and *SRD5A2* was performed. All coding exons and flanking intronic regions (±30 bp) of these seven DSD genes were sequenced using standard Sanger sequencing. In addition, Multiplex Ligation-dependent Probe Amplification was performed to detect larger deletions and duplications. Pathogenicity of detected variants was determined using Alamut Visual version 2.7.1 (Interactive Biosoftware, Rouen, France). Array-CGH was performed using CytoChip Oligo ISCA 8 × 60 K v2 array (with genome build numbers GRCh 36, 37, 38 and 39 with majority of cases being GRCh37). CNVs were validated using CytoChip Oligo ISCA 4 × 180 K. Duplications and deletions were confirmed by fluorescence *in situ* hybridization. Array-CGH analysis was performed using BlueFuse Multi v2.3 or v2.5 software (Illumina, Inc., San Diego, CA, USA) with CytoChip v2 algorithm. Any abnormal findings were compared with data held in the Database of Genomic Variants (http://dgv.tcag.ca/dgv/app/home?ref), DECIPHER (Cambridge, UK, https://decipher.sanger.ac.uk/) and the local database of variants. For all CNVs, the following databases were searched for information: NCBI including PubMed, OVID, Science Direct, Google Scholar, DECIPHER Ensembl Resources and GeneCards Human Gene Database. Search terms included the CNVs followed by ‘disorders of sex development’, ‘DSD’ and the phenotype of the boy from whom the CNVs was identified, for example ‘hypospadias’.

### Statistical analysis

Statistical analysis was performed with IBM SPSS Statistics 21 (Portsmouth, UK) and Minitab 17 Statistical Software Minitab, Inc. (Coventry, UK), and significance threshold was set at *P* < 0.05. Descriptive statistics were used to assess the distribution of data and all assumptions were assessed prior to inclusion of output. Mann–Whitney *U* tests were used for inter-group comparisons. To test association between variables, Pearson Chi-square test and linear-by-linear Chi-square test for trend were used.

## Results

### Cohort description

A total of 122 boys were identified with a presumed 46, XY karyotype (*n*, 42) or a definite 46, XY karyotype (*n*, 80). Median age at evaluation was 1.33 years (range 0.01–16.42) and this reflected the age at presentation to the specialist endocrine service. The phenotype of boys presenting to the service varied from isolated bilateral undescended testes (*n*, 41), isolated hypospadias (*n*, 33), isolated micropenis (*n*, 6) to a variable combination of all the above features (*n*, 42) (Supplementary Table S1). In the group of boys with isolated hypospadias, 25 were proximal, 4 were mid-shaft with the remaining 4 being distal and in the group of boys with a combination of features, 31 had hypospadias and of this group 26 had proximal hypospadias, 2 had mid-shaft and the remaining 3 boys had distal hypospadias. The median EMS of the cohort was 9 (1–11). An associated abnormality was identified in 39 (32%) boys with 14 (11%) having been diagnosed with a recognized syndrome (Supplementary Table S1). A family history of DSD was present in 16 (13%) boys and parental consanguinity was evident in 3 (2%). All 122 boys had endocrine investigations and 83 (68%) had an hCG stimulation test. Of the 122 boys, 76 (62%) also had genetic investigations with a total of 43 (35%) boys having array-CGH and 61 (50%) boys having gene panel analysis performed.

### Endocrine analysis

**Figure 1:**
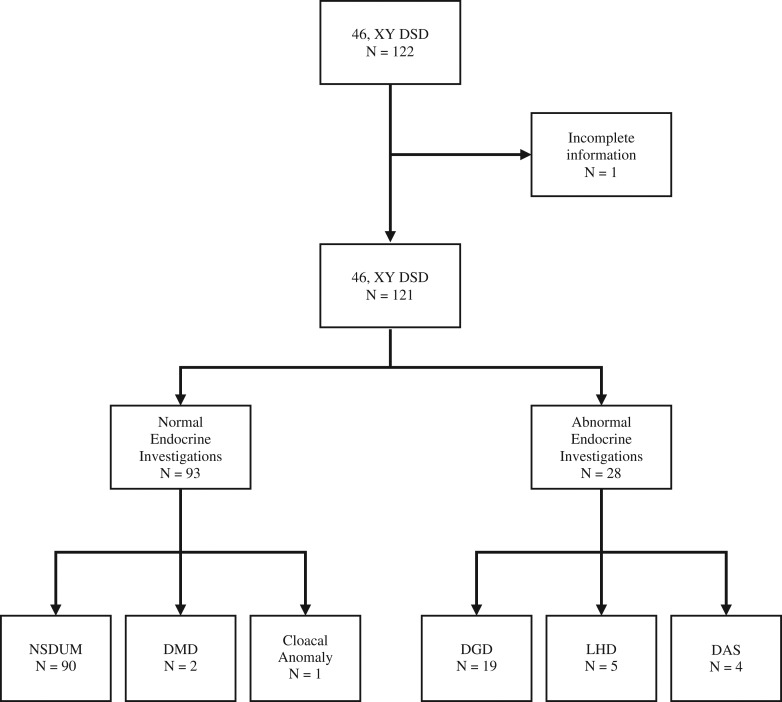
Consort diagram of 46, XY DSD boys who attended Glasgow DSD service between January 2010 and December 2015 for an assessment of atypical genitalia. Abbreviations: NSDUM, non-specific disorder of under-masculinization; DMD, disorder of Müllerian development; DGD, disorder of gonadal development; LHD, luteinising hormone deficiency; DAS, disorder of androgen synthesis.

Results of the hCG stimulation test were available for 83 boys with baseline androgen measurements available in an additional 38 boys. Of the 121 boys who had available results, an endocrine abnormality was present in 28 (23%) (Fig. 1). The median EMS of the boys with an endocrine abnormality and those without an endocrine abnormality was 8.3 (1–10.5) and 9 (1.5–11) (*P* = 0.028) respectively. Endocrine abnormalities pointed to a DGD in 19 (16%), luteinising hormone deficiency (LHD) in 5 (4%) and a DAS in 4 (3%). In the remainder who had normal endocrine investigations, there was one boy who had a cloacal anomaly, two (2%) boys who had a disorder of Müllerian development (DMD) and 90 (74%) boys had a non-specific disorder of under-masculinization (NSDUM) with normal gonadal function (Fig. 1).

### Genetic analysis

Of 43 boys (NSDUM, 30; DGD, 10; LHD, 3) who had array-CGH (Supplementary Table S1), CNVs were identified in 13 (30%) (NSDUM, 8; DGD, 5). The median EMS of the boys who had array-CGH performed was 9 (1.5–11); in those who did not have array-CGH testing, the EMS was also 9 (1–11). The median EMS of the boys who were found to have CNVs was 8.5 (1.5–11) compared to a median EMS of 9 (3–11) in the 30 boys who had a normal array-CGH (*P* = 0.598). One CNV was termed ‘likely pathogenic’ by the clinical diagnostic team, two CNVs were termed ‘likely benign’ and the CNVs in the remaining 10 boys were determined to be of ‘uncertain clinical significance’. Genes within the areas of deletions and duplications where CNV were identified are provided in Supplementary Table S2. Of these 43 boys who had array-CGH analysis, an associated abnormality was found in 11/13 (85%) of the boys in whom CNVs were identified and in 15/30 (50%) of boys who were found to have normal array-CGH (*P* = 0.033). Of the 61 boys (NSDUM, 41; DGD, 15; DAS, 2; DMD, 2; Cloacal Anomaly, 1) who had the limited seven-gene panel analysis, variants were identified in 6 (10%) (NSDUM, 3; DGD, 1; LHD, 2) with a median EMS of 6 (3, 9). The median EMS of the boys who had normal gene panel analysis was 9 (1.5–10) and was not significantly different to that in the boys where a gene variant was found (*P* = 0.312). There were three boys who had single gene analysis performed prior to availability of the gene panel in Glasgow, the median EMS of these boys was 8 (1–9) and all three boys had a single gene variant detected. For further details of genetic abnormalities identified, refer to the detailed section below and Table I.

**Table I:** Genetic abnormalities identified in 21 46, XY DSD boys.

Subject Ref	Endocrine result	EMS	Associated malformation	Single gene abnormality	CNV	Additional genetic tests performed
72	NSDUM	1.5	DD, Dysmorphic features	Not identified	Dup 7q36.3 - US	No
128	NSDUM	3	None	Compound Heterozygous *SRD5A2* c.[16 C > T];[680 G > A] p.[(Gln6*)];[(Arg227Gln)]	Not analysed	No
86	NSDUM	3	Capillary haemangioma on head, skull abnormality	Heterozygous *NR5A1* c.[1379 A > T];[=] p.[(Gln460Leu)];[(=)]	Dup 2p16.3 - US	No
82	NSDUM	6	Dysmorphic features and microcephaly	Not identified	Dup 16p11.2 - LB	No
24	NSDUM	9	None	Not analysed	Del 7q34 - US	No
25	NSDUM	9	None	Heterozygous *NR5A1* c.[185 G > T];[=] p.[(Arg62Leu)];[(=)]	Not analysed	No
56	NSDUM	9	LD, focal seizures	Not identified	Del 1q31.1, Del 5p14.3, Dup 13q32.1 - US	No
69	NSDUM	9	DD, speech delay	Not identified	Dup 15q11.1 - US	*FRAXA*—normal
121	NSDUM	9	LD, thickened soft tissues	Not analysed	Del 11p11.2 - US	N
99	NSDUM	11	DD, bilateral retinal coloboma, Right iris coloboma, visual impairment	Not analysed	Del 20p13 - US	N
65	DGD	3	Dysmorphic features, short stature, LD, panhypopituitarism	Not identified	Del 12q13.12 - US	N
28	DGD	6	DD, microcephaly and Fallot's tetralogy	Not analysed	Del 2p.22.3 - LB	22q11.2—normal
40	DGD	7.5	Encephalocoele, unilateral renal agenesis	Not identified	Del 4q13.3, Del 16p12.2, Dup 20p12.3	*NPHP1*—normal
91	DGD	8.5	DD, short stature	Not identified	Dep 18q21.32 - LP	No
132	DGD	9	None	Not identified	Dup 11q11q - US	No
90	DGD	9	Aspergers and Autism spectrum disorder	Heterozygous *NR5A1* c.[1019 C > T];[=] p.[(Ala340Val)];[(=)]	Not analysed	No
58	DAS	1	None	Compound heterozygous *SRD5A2* c.[698 + 1 G > T];[737 G > A] p.[(?)];[(Arg246Gln)]	Not analysed	No
38	DAS	3	None	Compound Heterozygous *SRD5A2* c.[698 + 1 G > T];[737 G > A] p.[(?)];[(Arg246Gln)]	Not analysed	No
45	DAS	8	None	*SRD5A2* (phase unknown) c.268 C > T(;)307 C > T p.(His90Tyr)(;)(Arg103*)	Not analysed	No
76	DAS	9	None	Heterozygous *SRD5A2* c.[548-2 A > C];[=] p.[(?)];[(=)]	Not analysed	No
1	DMD	9	None	Compound Heterozygous *AMH* c.[500 A > G]; [1669 T > A] p.[(Tyr167Cys)];[(Cys557Ser)]	Not analysed	No

Details of the genetic abnormalities identified in 46, XY DSD boys who were investigated by the Glasgow DSD service. Of the 76 boys who had genetic investigations performed, 20 boys were found to have either a single gene abnormality or CNV identified, and one boy had both a single gene and an array-CGH abnormality. Phenotypic information as well as the results of endocrine investigations are provided to demonstrate the variation in presentation of boys with genetic abnormalities identified.

Abbreviations: CNV, copy number variant; NSDUM, non-specific disorder of under-masculinization; DGD, disorder of gonadal development; DAS, disorder of androgen synthesis; DMD, disorder of Müllerian development; EMS, external masculinization score; DD, developmental delay; LD, learning difficulties; Dup, duplication; Del, deletion; US, uncertain clinical significance; LB, likely benign; LP, likely pathogenic.

### Disorders of gonadal development

Endocrine evaluation in 19 of 121 (16%) boys revealed a pattern consistent with a DGD. Median EMS of the 19 boys was 9 (3–10.5) and a family history of DSD was present in five cases. Of the 19 boys, 2 boys had bilateral anorchia. Median (range) serum AMH, FSH, LH and hCG-stimulated testosterone were 79.2 pmol/l (<0.4–268), 8.3 U/L (1.6–196.4), 4.8 U/l (1.1–39.1) and 0.8 nmol/l (<0.5–4.7), respectively. Additional abnormalities were found in nine (47%) boys with three boys having a diagnosis of a recognizable syndrome. Of the 19 boys, 10 had array-CGH and gene panel analysis with a further 5 cases having gene panel analysis only. Of the 10 cases who had array-CGH analysis, CNVs were identified in 5 (50%) and of the 15 cases that had gene panel testing, a pathogenic *NR5A1* variant was identified in 1 (7%) boy.

### Luteinizing hormone deficiency

Of 121 boys who had endocrine investigations, 5 (4%) boys with a median EMS of 8 (6–9) were found to be LH deficient. Median (range) serum AMH, LHRH-stimulated FSH, LHRH-stimulated LH and hCG-stimulated testosterone were 50.6 pmol/l (21.8–686), 4.3 U/l (<0.1–8.6), 0.9 U/l (<0.1–2.3) and 0.7 nmol/l (<0.5–1.0), respectively. Of these five boys, four had associated abnormalities. A diagnosis of CHARGE syndrome was made in three boys, with mutations in *CHD7* found in two of these boys. There was no family history of DSD for any of these five boys. Genetic investigations were performed in three boys. All three had array-CGH performed with two boys having additional gene panel analysis and all of these genetic investigations were found to be normal in all three boys.

### Disorder of androgen synthesis

The median EMS of the four boys diagnosed with 5α-reductase type 2 deficiency (5-ARD) was 4 (1, 9) and a family history of DSD was known in two boys. There were no associated abnormalities in this group. Pathogenic *SRD5A2* variants were identified in two boys who had single gene sequencing due to a family history of 5-ARD. In addition, pathogenic *SRD5A2* variants were also identified in two boys who had routine panel testing without any clear evidence of biochemical abnormalities at evaluation. In one of these boys, urinary steroid analysis by GCMS had not identified an endocrine abnormality on initial testing when performed at the age of 2 days, however, when repeated at the age of 6 years following molecular confirmation of 5-ARD, urinary steroid analysis was clearly abnormal.

### Disorder of Müllerian development

Of the 93 boys who had normal biochemistry, 2 boys with a median EMS of 10 (9–11) were found to have Müllerian structures present on ultrasound investigation and surgical exploration. Neither of these boys had other associated abnormalities or a family history of DSD. One boy underwent targeted DNA analysis for variants in *AMH*, which revealed compound heterozygous mutations.

### Non-specific disorder of under-masculinization

The largest subgroup of boys identified included boys who had normal endocrine investigations. Of these 90 boys with NSDUM, a 46, XY karyotype was confirmed in 66 (73%) boys and the median EMS was 9 (1.5–11). Associated abnormalities were identified in 26 (29%) boys with a recognized syndrome being diagnosed in 8 (9%). In 2 of the 90 (2%) boys, there was a positive history of parental consanguinity, and a family history of DSD was identified in 11 (12%). Array-CGH was performed in 33 boys and a CNV was detected in 10 (30%) of cases. A likely benign CNV was identified in one boy and the remaining nine cases were deemed to be of uncertain clinical significance. Gene panel analysis was performed in 45 (50%) boys, with a pathogenic variant detected in *SRD5A2* in 1 boy and a pathogenic variant detected in *NR5A1* in 2 boys.

### Predicting the likelihood of identifying a genetic or endocrine abnormality

The presence of an associated abnormality was significantly associated with the likelihood of detecting CNVs on array-CGH analysis (*P* = 0.033) since 11/26 (43%) of these cases who had array-CGH also had an associated microdeletion or microduplication. Abnormal endocrine investigations, however, were not a predictor of detecting a CNVs. In the 13 boys who had CNVs identified, 5 (38%) were found to have abnormal endocrine investigations, and in the 30 boys who had normal array-CGH results, 8 (37%) were found to have abnormal endocrine investigations (*P* = 0.439). EMS was not significantly associated with detection of CNVs with a median EMS of 8.5 (1.5, 11) in the boys who had a CNV detected and 9 (3–11) in the group who had a normal array-CGH (*P* = 0.596). The likelihood of identifying pathogenic variants on gene panel analysis in those cases with abnormal biochemistry (*P* = 0.26), associated abnormality (*P* = 0.475) or a low EMS (*P* = 0.2) did not reach statistical significance. The median EMS of boys who had normal endocrine investigations was 9 (1.5–11) and was higher than that in boys who had an endocrine abnormality at 8.3 (1–10.5) (*P* = 0.028).

## Discussion

This study describes in detail the likelihood of detecting an endocrine or a genetic abnormality in a cohort of 46, XY boys who present to a multidisciplinary service for an assessment of atypical genitalia. In this cohort, endocrine investigations were abnormal in about a quarter whilst genetic analysis revealed an array-CGH abnormality in 30% and a single gene disorder in almost 15%. This large, well-characterized group of boys also highlighted the diverse presentations of boys with atypical genitalia with almost a third of boys having an additional malformation and similar to what has been reported in large multicentre cohorts (Cox *et al.*, 2014). Neurocognitive abnormalities are often associated with DSD (Gazdagh *et al.*, 2016) and this was also observed in the current study.

The comprehensive approach used in the current study has highlighted that genetic abnormalities and endocrine abnormalities do not always associate with each other. Over three-quarters of boys investigated had normal endocrine investigations and of those who also had molecular genetics and array-CGH analysis, a genetic abnormality was found in 20%. In those with an abnormal endocrine investigation, over two-thirds had an abnormality consistent with a primary DGD. Of this group of 19 boys with a DGD, only one had a molecular genetic abnormality whilst 50% of those who had array-CGH displayed a CNV. On the other hand, of the rest of the boys who had normal endocrine investigations, 30% of those who had array-CGH displayed a CNV and about 10% of boys who had a limited gene panel had an abnormal finding.

The overall CNV detection rate of 30% in this group of boys is comparable to previous reports (Tannour-Louet *et al.*, 2010; Baetens *et al.*, 2014). As expected, the presence of an associated abnormality was a strong predictor of identifying a CNV, emphasizing the value of careful examination for the presence of associated malformations in boys with 46, XY DSD. Three-quarters of the CNVs identified in this cohort were clinically classified as being of uncertain significance, however, on detailed database review half had been previously reported in cases associated with a DSD. This mismatch highlights the need for diagnostic services to allocate further expert resources to interpretation of genetic findings.

The overall yield of molecular genetic abnormalities of approximately 15% of boys was lower than previous reports (Eggers *et al.*, 2016; Alhomaidah *et al.*, 2017). However, the likelihood of identifying a gene defect may be influenced by the strategy that is adopted for undertaking molecular genetic analysis. For instance, a targeted gene analysis approach may yield more variants when selecting cases that have a clear defect of androgen synthesis (Morel *et al.*, 2011; Eggers *et al.*, 2016). Identification of a genetic abnormality in *AR* may have been higher in the past when cases were selected by studying AR binding (Ahmed *et al.*, 2000a). A recent study in boys with 46, XY DSD also suggests that the likelihood of finding a genetic mutation may also depend on the prevalence of consanguinity (Ozen *et al.*, 2017). However, the current study clearly shows that molecular genetic abnormalities may also be evident in cases with apparently normal androgen synthesis. Disorders of testosterone biosynthesis such as 17β-hydroxysteroid dehydrogenase type 3 (17β-HSD3) deficiency or 5α-reductase type two deficiency (5-ARD) are characterized by a reduction in conversion of androstenedione (A) to testosterone (T) or testosterone to dihydrotestosterone (DHT), respectively. Typically, these conditions are associated with an alteration in the ratio of the two forms of androgens so that 17β-HSD3 deficiency is associated with a low T:A ratio (Ahmed *et al.*, 2000c) and 5-ARD is associated with a high T:DHT ratio (Saenger *et al.*, 1978). However, a low T:A ratio may not be very sensitive (Lee *et al.*, 2007) or specific (Ahmed *et al.*, 2000c) for 17β-HSD3 deficiency and the T:DHT ratio may not be sufficiently sensitive for identifying cases of 5-ARD (Ng *et al.*, 2000; Maimoun *et al.*, 2011). The hCG stimulation test which is frequently used to assess androgen synthesis and testicular function in prepubertal boys remains a controversial test when it comes to defining a normal response, given that the response may vary depending on the regimen and the developmental age of the child (Dixon *et al.*, 2007). Furthermore, molecularly confirmed cases of androgen insensitivity syndrome who would be expected to have normal androgen synthesis may show a poor response on hCG stimulation (Ahmed *et al.*, 1999). Others have suggested that selecting cases by the extent of under-masculinization may lead to a higher likelihood of genetic abnormalities (Su *et al.*, 2015). However, the current study shows that a large proportion of cases with a genetic abnormality were not particularly under-masculinized.

A notable limitation of the study was the evolution of the strategy for genetic analysis over the study period. Although the endocrine evaluation of this cohort followed as much as possible the guidance proposed by recent expert groups (Ahmed *et al.*, 2016), the extent of genetic investigations which were performed in this group of boys was variable and dependent on the availability of the genetic tests. The seven-gene panel for XY DSD became available in Glasgow in 2014 with a subsequent lowering of threshold for genetic investigations. Similarly, the availability of array-CGH increased in recent years with testing often being performed where there were associated abnormalities. It is clear that variation in several other candidate genes may lead to XY DSD (Eggers *et al.*, 2016; Alhomaidah *et al.*, 2017) and the gene panel reported in this study has its obvious limitations. With respect to molecular genetic testing, our strategy has been to incorporate the genetic testing into our clinically accredited hospital diagnostic laboratory rather than a research laboratory and as recommended by the UK DSD taskforce (Ahmed *et al.*, 2016). This strategy is expected to extend to the development of a larger panel of genes as expert centres increasingly adopt Next Generation Sequencing (NGS) technology for routine diagnostics (Baxter *et al.*, 2015; Eggers *et al.*, 2016; Ozen *et al.*, 2017).

In addition, the algorithmic approach that has often been recommended in the past when genetic tests were performed to confirm the biochemical findings (Hughes *et al.*, 2012; Ahmed *et al.*, 2013) may also change. It is increasingly debatable whether this stepwise approach remains appropriate. In a recent survey of expert centres in the I-DSD Registry, approximately half of the respondents opted for a genetic rather than a biochemical test for diagnosing 5-ARD and 17β-HSD deficiency and many would pursue a genetic diagnosis irrespective of the results of the endocrine investigations (Kyriakou *et al.*, 2016). Whilst there are clear perceived advantages of adopting a non-selective NGS-based approach with identification of a larger number of genetic variants (Eggers *et al.*, 2016), a concern remains about the functional relevance of these genetic variants identified subsequently and it is possible that, in the future, the place for biochemical evaluation may be to confirm or refute the functional relevance of a molecular genetic finding as well as for long-term monitoring following a genetic diagnosis. Given that a number of cases may have an endocrine abnormality but no clear genetic abnormality, perhaps the most pragmatic approach is to perform both endocrine and genetic investigations in parallel, rather than in a stepwise manner but within the framework of an expert multidisciplinary diagnostic team (Alhomaidah *et al.*, 2017).

## Supplementary data

Supplementary data are available at *Human Reproduction* online.

## Supplementary Material

Supplementary Data

Supplementary Data
