# Supplementary material for: Prevalence of endocrine and genetic abnormalities in boys evaluated systematically for a disorder of sex development
Source: Hum Reprod. 2017 Aug 30;32(10):2130–7. doi: 10.1093/humrep/dex280 (PMC5850224; doi:10.1093/humrep/dex280)
Supplement: Supplementary Data [file dex280suppl_table1.pdf]

Human Reproduction, 2017
doi:10.1093/humrep/dex280

human reproduction

SUPPLEMENTARY DATA

Supplementary Table SI **Detailed phenotypic information and results for all 46, XY DSD boys who had array-CGH performed as part of an investigation for atypical genitalia. Details of HGNC genes identified within CNV region are included in Supplementary Table 2.**

Patient No.	Undescended testes	Hypospadias	Micropenis	Bifid scrotum	EMS	Associated abnormality	Family history	CNV
91	Unilateral	Distal	No	No	8.5	DD, short stature	None	18q21.32
24	Bilateral	None	Yes	No	8	None	None	7q34
40	Unilateral	Proximal	No	No	7.5	Encephalocoele, unilateral renal agenesis	Yes	4q13.3
56	No	None	Yes	No	9	LD, focal seizures	None	1q31.1, 5p14.3, 13q32.1
65	Bilateral	Proximal	Yes	No	3	Dysmorphic features, short stature, LD, panhypopituitarism	Yes	12q13.12
69	No	Proximal	No	No	9	DD, speech delay	None	15q11.1
72	Unilateral	Proximal	Yes	Yes	1.5	DD, dysmorphic features	None	7q36.3
86	Bilateral	Proximal	No	Yes	3	Capillary haemangioma, skull abnormality	Yes	2p16.3
99	Bilateral	None	No	No	11	DD, bilateral retinal coloboma, R iris coloboma, visual impairment	None	20p13
121	No	None	Yes	No	9	LD, thickened soft tissues	None	11p11.2
132	Bilateral	None	No	No	9	None	None	11q11
28	Bilateral	None	Yes	No	6	DD, microcephaly and Fallot's tetralogy	Yes	2p.22.3
82	No	Proximal	No	Yes	6	Dysmorphic features and Microcephaly	None	16p11.2
9	No	Proximal	No	No	9	Dysmorphic features, A-R malformation	None	None
10	Bilateral	Proximal	No	No	6	None	None	None
12	No	None	Yes	No	9	None	None	None
13	Bilateral	Proximal	Yes	No	3	None	Yes	None
19	Unilateral	Mid-shaft	No	No	9	L choanal atresia, accessory nipple, second and third toe syndactyly	None	None
22	Bilateral	None	Yes	No	7	None	None	None
23	No	Mid-shaft	Yes	No	7	Limb asymmetry	None	None
26	Bilateral	None	Yes	No	7	Fallot's tetralogy, bilateral retino-choroidal coloboma, mixed astigmatism, nystagmus, bilateral hearing loss, DD (CHARGE syndrome)	None	None
29	No	Proximal	Yes	No	6	Chest wall/limb asymmetry, epicanthic folds R > L	None	None
30	Bilateral	None	No	No	9	None	None	None
31	Unilateral	Proximal	No	No	9	None	None	None
33	Bilateral	None	No	No	9	Motor delay, hypotonia, bilateral congenital hydronephrosis, laryngomalacia, café au lait patches, hyperteloric with ptosis and plagiocephaly. (Neurofibromatosis type 1—NF1)	None	None
35	No	Distal	No	No	11	None	Yes	None
39	Bilateral	None	No	No	9	None	None	None
43	No	Proximal	No	Yes	6	None	None	None
44	Bilateral	None	No	No	9	LD, sensorineural deafness, dysmorphic features	None	None
46	Bilateral	None	No	No	10	None	Yes	None
47	Bilateral	None	No	No	9	Mitral regurgitation, PUJ obstruction, (Noonan syndrome)	None	None

Continued

Supplementary Table SI *Continued*

Patient No.	Undescended testes	Hypospadias	Micropenis	Bifid scrotum	EMS	Associated abnormality	Family history	CNV
48	Bilateral	None	No	No	9	None	None	None
54	Bilateral	None	No	No	9	None	None	None
70	Unilateral	Proximal	No	No	7.5	DD	None	None
73	Bilateral	Proximal	Yes	No	5	None	None	None
77	Bilateral	Proximal	No	Yes	4	None	None	None
83	Bilateral	None	Yes	No	6	DD	None	None
92	Bilateral	None	No	No	9	Dystonic quadriplegic cerebral palsy, hearing impairment, communication difficulties, breathing problems.	None	None
95	Bilateral	None	No	No	9	NK	NK	None
106	No	Proximal	Yes	Yes	3	None	None	None
126	No	Proximal	Yes	No	6	None	None	None
129	Bilateral	None	Yes	No	6	None	None	None
130	Bilateral	None	Yes	No	6	Microcephaly, juvenile arthritis	None	None
136	Bilateral	None	Yes	No	6	None	None	None

Abbreviations: HGNC, HUGO Gene Nomenclature Committee; CNV, copy number variant; CGH, comparative genomic hybridization; DD, developmental delay; A-R, anorectal; LD, learning difficulties; NK: not known.
