# Supplementary material for: Prevalence of endocrine and genetic abnormalities in boys evaluated systematically for a disorder of sex development
Source: Hum Reprod. 2017 Aug 30;32(10):2130–7. doi: 10.1093/humrep/dex280 (PMC5850224; doi:10.1093/humrep/dex280)
Supplement: Supplementary Data [file dex280suppl_table2.pdf]

Supplementary Table SII **Details of HGNC Genes identified within CNVs in 46, XY DSD boys. Information was not available for cases 72 and 121.**

Subject Ref	CNV	Protein coding genes	Non-protein coding genes
24	Del 7q34	*MTRNR2L6, PRSS1*	*PGBD4P1, TRBV10-1, TRBV11-1, TRBV12-1, TRBV19, TRBV20-1, TRBV21-1, TRBV22-1, TRBV23-1, TRBV24-1, TRBV 25-2, TRBV26, TRBV27, TRBV28, TRBV29-1, TRBV5-2, TRBV5-3, TRBV6-4, TRBV7-3, TRBV8-1, TRBV8-2, TRBV9, TRBVA, TRBVB*
28	Del 2p.22.3	Does not span any genes	Does not span any genes
40	Del 4q13.3	*ADAMTS3, NPFFR2*	*HNRNPA1P67*
	Del 16p12.2	*IGSF6, METTL9, NPIPB4, OTOA,*	*RNU6-1005P, RNU6-196P, RRN3P1*
	Dup 20p12.3	*PLCB1, PLCB4*	
56	Del 1q31.1	Does not span any genes	Does not span any genes
	Del 5p14.3	*CDH18*	*GUSBP1*
	Dup 13q32.1	*FARP1, HS6ST3, IPO5, MBNL2, OXGR1, RAP2A, RNF113B, STK24*	*FARP1-AS1, FTLP8, HSP90AB6P, LINC00359, LINC00456, MIR3170, PSMA6P4, RN7SKP7, RN7SKP8, RNA5SP37, RPL7AP61, TULP3P1*
65	Del 12q13.12	*AQP2, AQP5, AQP6, BCDIN3D, FAIM2, FAM186B, FMNL3, KCNH3, MCRS1, NCKAP5L, PRPF40B, RACGAP1, SPATS2, TMB1M6,*	*BCDIN3D-AS1, HIGD1AP9, LSM6P2, POLR2KP1, RNU6-834P*
69	Dup 15q11.1	*CYFIP1, GOLGA6L1, GOLGA6L2, GOLGA6L6, MKRN3, NIPA1, NIPA2, OR4M2, OR4N4, POTEB, POTEB2, TUBGCP5*	*ABCB10P1, BMS1P16, CT60, CXADRP2, ELMO2P1, GOLGA8CP, GOLGA8DP, GOLGA8EP, GOLGA8I, GOLGA8S, GRAMD4P5, GRAMD4P6, HERC2P2, HERC2P3, HERC2P7, IGHD1OR25-1B, IGHD2OR15-2B, IGHD3OR15-3B, IGHD4OR15-4B, IGHD5OR15-5B, IGHV1OR15-1, IGHV1OR15-3, IGHV1OR15-4, IGV1OR15-6, IGHV4OR15-8, KIAA0125P2, MIR1268A, MIR3118-4, MIR3118-6, MIR4508, MIR4509-1, MIR5701-1, MIR5701-2, NBEAP1, OR11H3P, OR11J1P, OR11J2P, OR11J5P, OR11K1P, OR4H6P, OR4N3P, OR4Q1P, RN7SL106P, RN7SL400P, RN7SL495P, RN7SL536P, RN7SL545P, RN7SL759P, RNU6-1235P, RNU6-498P, RNU6-631P, RNU6-749P, SPATA31E2P, SPATA31E3P, TUBGCP5, WHAMMP3*
82	Dup 16p11.2	Does not span any genes	Does not span any genes
86	Dup 2p16.3	*FSHR, GTF2A1L, LHCGR, STON1, STON1-GTFA1L, TCEB2P3, TPT1P11*	*CTBP2P5, RNU6-439P, STON1-GTFA1L, TCEB2P3, TPT1P11*
91	Dep 18q21.32	*ADNP2, ALPK2, ATP9B, BCL2, C18of63, CBLN2, CCBE1, CCDC102B, CD226, CDH19, CDH20, CDH7, CNDP1, CNDP2, CPLX4, CTDP1, CYB5A, DOK6, DSEL, FAM69C, FBXO15, GALR1, GRP, HMSD, HSBP1L1, KCNG2, KDSR, KIAA1468, LMAN1, MALT1, MBP, MC4R, NETO1, NFATC1, PARD6G, PHLPP1, PIGN, PMAIP1, PQLC1, RAX, RBFA, RNF152, RTTN, SALL3, SEC11C, SERPINB11, SERPINB12, SERPINB13, SERPINB1, SERPINB2, SERPINB3, SERPINB4, SERPINB5, SERPINB7, SERPINB8, SIMIM21, SOCS6, TIMM21, TMX3, TNFRSF11A, TSHZ1, TXNL4A, VPS4B, ZADH2, ZCCHC2, ZNF236, ZNF407, ZNF516, ZNF532*	*ACTBP9, AKR1B10P2, ARL2BPP1, ATP5G1P6, ATP9B, BDP1P, CTBP2P3, FAM60CP, FAUP1, GLUD1P4, GTSCR1, HNRNPA1P11, LINC00305, LINC00683, LINC00908, LINC00909, LINC01029, MIR5011, MIR548AV, MRPL37P1, MRPS5P4, NFE2L3P1, OACYLP, PRPF19P1, RBFADN, RN7SL112P, RN7SL342P, RN7SL401P, RN7SL551P, RN7SL705P, RN7SL795P, RNA5SP461, RNU2-69P, RNU4-17P, RNU6-1037P, RNU6-116P, RNU6-142P, RNU6-219P, RNU6-346P, RNU6-39P, RNU6-567P, RNU6-655P, RNU7-146P, RPIAP1, RPL12P39, RPL17P44, RPL26P35, RPL30P14, RPL31P9, RPL9P31, RPS2P6, RPS3AP49, SDCCAG3P1, SDHCP1, SERPINB10, SLC25A6P4,*
99	Del 20p13	*ANGPT4, C20orf202, C20ort96, CSNK2A1, DEFB125, DEFB126, DEFB127, DEFB128, DEFB129, DEFB132, FAM110A, FKBP1A, NRSN2, NSFL1C, PSMF1, RAD21L1, RBCK1, RSPO4, SCRT2, SCRT2, SDCBP2, SIRPB1, SIRPB2, SIRPD, SIRPG, SLC52A3, SNPH, SOX12, SRXN1, TBC1D20, TCF15, TMEM74B, TRIB3, ZCCHC3,*	*ACTG1P3, RN7SL561P, RNU6-917P, SDCBP2-AS1, SIRPB3P,*
132	Dup 11q11q	*OR10AG1, OR4A15, OR4A16, OR4C11, OR4C15, OR4C16, OR4C6, OR4P4, OR4S2, OR5AS1, OR5D13, OR5D14, OR5D16, OR5D18, OR5F1, OR5I1, OR5J2, OR5L1, OR5L2, OR5W2, OR8H2, OR8K3, OR8I2, OR8J2, OR8J3, OR8K5, TRIM51*	*OR10AF1P, OR10AK1P, OR4A10P, OR4A11P, OR4A13P, OR4A14P, OR4A17P, OR4A21P, OR4A50P, OR4A9P, OR4C11, OR4C14P, OR4P1P, OR4V1P, OR4X7P, OR5AQ10, OR5BE1P, OR5BN1P, OR5BN2P, OR5D17P, OR5D2P, OR5D3, OR5F2P, OR5J1P, OR5J7P, OR5W1P, OR7E5P, OR8H3, OR8I4P, OR8K4P, OR8V1P, OR9M1P*

Abbreviations: HGNC, HUGO Gene Nomenclature Committee; CNV, copy number variants; Dup, duplication; Del, deletion.
